# Dietary magnesium, C-reactive protein and interleukin-6: The Strong Heart Family Study

**DOI:** 10.1371/journal.pone.0296238

**Published:** 2023-12-21

**Authors:** Nandana D. Rao, Rozenn N. Lemaitre, Colleen M. Sitlani, Jason G. Umans, Karin Haack, Veronica Handeland, Ana Navas-Acien, Shelley A. Cole, Lyle G. Best, Amanda M. Fretts

**Affiliations:** 1 Institute of Public Health Genetics, University of Washington, Seattle, Washington, United States of America; 2 Department of Medicine, University of Washington, Seattle, Washington, United States of America; 3 Cardiovascular Research Health Unit, University of Washington, Seattle, Washington, United States of America; 4 MedStar Health Research Institute, Hyattsville, Maryland, United States of America; 5 Department of Medicine, Georgetown University, Washington, DC, United States of America; 6 Texas Biomedical Research Institute, San Antonio, Texas, United States of America; 7 Indian Health Services, Rockville, Maryland, United States of America; 8 Department of Environmental Health Science, Columbia University, New York, New York, United States of America; 9 Missouri Breaks Industries Research Inc, Eagle Butte, South Dakota, United States of America; 10 Department of Epidemiology, University of Washington, Seattle, Washington, United States of America; Mexican Social Security Institute, MEXICO

## Abstract

**Objectives:**

To examine the associations of dietary Mg intake with inflammatory biomarkers (C-reactive protein (CRP) and interleukin 6 (IL-6)), and the interaction of dietary Mg intake with single nucleotide polymorphism (SNP) rs3740393, a SNP related to Mg metabolism and transport, on CRP and IL-6 among American Indians (AIs).

**Methods:**

This cross-sectional study included AI participants (n = 1,924) from the Strong Heart Family Study (SHFS). Mg intake from foods and dietary supplements was ascertained using a 119-item Block food frequency questionnaire, CRP and IL-6 were measured from blood, and SNP rs3740393 was genotyped using MetaboChip. Generalized estimating equations were used to examine associations of Mg intake, and the interaction between rs3740393 and dietary Mg, with CRP and IL-6.

**Results:**

Reported Mg intake was not associated with CRP or IL-6, irrespective of genotype. A significant interaction (p-interaction = 0.018) was observed between Mg intake and rs3740393 on IL-6. Among participants with the C/C genotype, for every 1 SD higher in log-Mg, log-IL-6 was 0.04 (95% CI: -0.10 to 0.17) pg/mL higher. Among participants with the C/G genotype, for every 1 SD higher in log-Mg, log-IL-6 was 0.08 (95% CI: -0.21 to 0.05) pg/mL lower, and among participants with the G/G genotype, for every 1 SD higher in log-Mg, log-IL-6 was 0.19 (95% CI: -0.38 to -0.01) pg/mL lower.

**Conclusions:**

Mg intake may be associated with lower IL-6 with increasing dosage of the G allele at rs3740393. Future research is necessary to replicate this finding and examine other Mg-related genes that influence associations of Mg intake with inflammation.

## Introduction

Magnesium (Mg) is a critical element in human nutrition, involved in over 600 enzymatic reactions and multiple metabolic pathways that influence a myriad of physiologic systems [1]. Major sources of dietary Mg include leafy green vegetables, nuts, seeds, and whole grains [2]. Observational studies suggest that individuals who consume diets high in dietary Mg have lower levels of inflammation as evaluated by C-reactive protein (CRP) concentrations when compared to individuals whose diets are low in Mg [3–5]. On the other hand, results from randomized controlled trials (RCTs) that tested the impact of oral Mg supplementation on inflammation demonstrate mixed results; some RCTs show no impact of oral Mg supplementation on CRP levels [6–10], while a large meta-analysis of RCTs suggests that supplementation reduces CRP levels [11]. Most published studies have focused on CRP alone, and the relationship of dietary Mg and interleukin-6 (IL-6), another marker of inflammation, is unclear [3, 12, 13].

Several biological mechanisms may in part explain the relationship of Mg and inflammation. Both animal and in vitro studies suggest that lower levels of Mg increase intracellular Ca^2+^, a signal thought to initiate inflammatory responses by cells [1]. Genetics may also play a role in the body’s ability to properly utilize dietary Mg. Genes associated with abnormal Mg metabolism and Mg transport have been identified [14, 15], but whether these loci influence the relationship of dietary Mg intake with inflammation is largely unknown. Better understanding the potential interactions of genetic loci associated with Mg metabolism and dietary Mg intake on inflammatory markers may provide further insight about potentially relevant biological mechanisms.

The purpose of this study was to examine the associations of reported intake of dietary Mg with inflammatory biomarkers (CRP and IL-6) among American Indians (AI) who participated in the Strong Heart Family Study (SHFS). Additionally, we examined potential interactions of Mg intake with the single nucleotide polymorphism (SNP) rs3740393. This SNP is located in *CNNM2*, a gene thought to play an important role in Mg homeostasis and transport; the SNP showed a significant association (p = 8.58E-07) with serum Mg concentrations in a previous meta-analysis of genome wide association studies from the CHARGE consortium [16]. Given the high prevalence of diabetes and cardiovascular diseases (CVD) among AIs, it is important to understand whether participants who consume more Mg have lower levels of inflammation than participants whose diets are lower in Mg, and whether the association of dietary Mg with markers of inflammation varies according to genotype. This information may help inform nutritional guidelines and diabetes and CVD prevention strategies in this at-risk population.

## Materials and methods

### Study setting

The SHFS is a study of CVD and its risk factors among 12 AI communities located in Arizona, North Dakota, South Dakota, and Oklahoma. The cohort completed two study examinations (2001–2003 and 2007–2009) over eight years. Both study exams included a personal interview, physical examination, medication review, laboratory work-up, and dietary assessment. Details on the study methods and data collection instruments have been reported previously [17]. In total, 1,122 men and 1,658 women took part in the study. Institutional review boards from each Indian Health Service region in Arizona, North Dakota, South Dakota, and Oklahoma and all participating communities approved the study. Written informed consent was obtained from all participants. Tribal communities were also involved in all components of the study, including conceptualization, study design, development of data collection instruments, data collection, analysis, and dissemination. This research was performed in accordance with the ethical standards laid down in the 1964 Declaration of Helsinki.

### Study participants

The analytic sample included SHFS participants with available data on demographics, diet, and markers of inflammation (i.e., CRP and IL-6) at the baseline study exam (2001–2003). S1 Fig details participant inclusion for analyses. Those with incomplete family identification (n = 17), missing CRP (n = 421) or IL-6 (n = 156) measurements, or with unreliable dietary data were excluded from analysis. This included participants who did not complete the dietary assessment (n = 117), skipped more than 15% of the dietary assessment questions (n = 31), or who reported extreme caloric intake [intakes of <600 kcal/day or >6000 kcal/day for women (n = 73) and <600 or >8000 kcal/day for men (n = 41) [18]]. In total, 1,924 participants comprised the study sample for analyses that assessed the associations of dietary Mg intake with CRP and IL-6. For analyses that examined the interaction of Mg-related SNPs with dietary intake of Mg on CRP and IL-6, we additionally excluded participants missing genotype information (n = 338); in total, 1,586 participants comprised the study sample for genetic analyses.

### Dietary assessment

Average food intake over the past year was measured using an interviewer-administered Block food frequency questionnaire (FFQ) with ethnic foods supplement, as previously described [18, 19]. Participants were asked how often, on average, they consumed a particular food and portion size. In addition to the 119 items included on the standard Block FFQ, participants were also asked about the intake of foods commonly consumed in some participating communities including menudo, pozole, guysava, red or green chili, Indian tacos, frybread, and Spam. Use of multivitamins and single vitamins was also ascertained as part of the FFQ. Average daily energy and nutrient intakes, including dietary Mg, were calculated for each participant for all foods and vitamins (and then summed) using the Block database (Block Dietary Systems, Berkeley, CA) [18].

### Genotype assessment

Participants were genotyped using the Illumina Cardio-Metabo DNA Analysis BeadChip (MetaboChip), as described previously [20]. Of the SNPs genotyped, rs3740393 was selected for gene x diet interaction analysis and allele C was coded as the risk allele. This SNP was previously identified as related to Mg transport in relation to markers of inflammation (CRP and IL-6) [16]. Prior genetic studies across various ancestral groups have also found associations between SNPs rs1205 and rs3091244 with serum CRP [21–24]. These SNPs were selected as additional covariates for the gene x diet analysis for the outcome CRP in order to reduce the variance of CRP and improve sensitivity. For SNP rs1205, allele C was coded as the risk allele, while allele A was coded as the risk allele for SNP rs3091244.

### Assessment of CRP & IL-6

Blood samples were collected at the baseline exam after a 12-hour fast and circulating levels of CRP were assessed using an immunoturbidometric method (Vitros Chemistry Products, number 6801739, Ortho Clinical Diagnostics, Rochester, NY) on a Vitros 5,1 platform (Ortho Clinical Diagnostics, Rochester, NY). The sensitivity of the assay was 0.175 mg/L and the CV was less than 4.1%. The IL-6 assay was performed on the Bioplex 100 (Luminex, X-MAP) instrument and had an intra-assay CV of 3.51% and inter-assay CV of 4.48%.

### Other covariates

The baseline exam also included a standardized personal interview, physical exam, medication review, and a one-week pedometer log, as described previously [17]. Anthropometric measures were obtained with the participant wearing lightweight clothing and no shoes. Body mass index (BMI) was calculated as body weight divided by height-squared (kg/m^2^). Based on the American Diabetes Association criteria, any participant taking insulin or oral anti-diabetic medications at the baseline exam, or with fasting plasma glucose ≥ 126 mg/dl was considered to have diabetes [25]. Participants with prevalent CVD included those who had ever experienced a myocardial infarction or stroke, and/or had previously been diagnosed with coronary heart disease or congestive heart failure based on data collected at the personal interviews and medical record review.

### Statistical analyses

Generalized estimating equations (GEE) with an independence working correlation structure and robust standard errors were used to examine the cross-sectional relationship between dietary Mg and CRP, dietary Mg and IL-6, and to examine the role of gene x diet interactions in these relationships. GEE was selected to accommodate potential familial correlation within the data. All statistical analyses were conducted using R version 3.5.1.

Log-Mg intake was examined both continuously and categorically using quintiles. Quintiles were defined according to total Mg intake relative to total calorie intake, and the lowest quintile was used as the reference group. All analyses were adjusted for total calorie intake in order to avoid false associations of nutrients with inflammation due to over-or-under-reporting of dietary intake and differences in energy intake due to body size. All covariates were selected a priori based on potential associations with both dietary Mg intake and/or markers of inflammation as based on previously published literature [3–5]. Due to skew, Mg, CRP and IL-6 were log transformed (natural log scale) to decrease the influence of outliers.

Two models were fit to examine the association of Mg with CRP and IL-6. A crude model (Model A) adjusted for age, sex, site, and total calorie intake. Our primary model (Model B) additionally adjusted for education (y), smoking (packs per y), alcohol use (never, ever, current), pedometer-determined physical activity (steps per day), BMI (kg/m^2^), diabetes (yes/no), hypertension (yes/no), CVD (yes/no), and dietary factors including fiber (g per day), folate (mcg per day), total fat (% of calories), and fruits and vegetables (servings per day).

Given the high prevalence of diabetes and CVD in this population and the high levels of inflammation present among participants with these diseases, we conducted sensitivity analyses that excluded participants with prevalent diabetes and CVD. In consideration of individuals who may have been experiencing an acute infection, further sensitivity analyses excluded participants with CRP greater than 16 mg/L (+1 SD) or IL-6 greater than 16 pg/mL [26, 27]. In addition, we assessed potential interaction of reported Mg intake with age, sex, and BMI on CRP and IL-6 in exploratory analyses to investigate whether these factors modify the association of Mg intake with CRP or IL-6.

We examined the associations of SNPs rs3740393, rs1205, and rs3091244 with CRP and IL-6, controlling for age and sex. Additionally, we assessed the interaction of reported Mg intake with rs3740393 on levels of CRP and IL-6 by including an interaction term (Mg x rs3740393) in a model also adjusted for Mg, rs3740393, and crude model (Model A) or primary model (Model B) covariates. For the outcome of CRP, Model B further adjusted for SNPs associated with CRP levels (i.e., rs1205, rs3091244).

Multiple imputation was used to fill in occasional missing values of covariates (<3% for all covariates) in order to conserve maximum sample size. This imputation was implemented using the predictive mean matching method with the MICE package in R. Imputed values were predicted across 10 datasets using age, sex, site, total calorie intake, alcohol use, and other dietary variables.

## Results

The average age of study participants was 40.3 years and 39% were male. Median (IQR) dietary Mg intake was 283.1 (198.4–407.9) mg/day. There were 441 participants (22.9%) who reported taking Mg supplements or multivitamins containing Mg. Median (IQR) CRP levels were 3.7 (1.5–7.9) mg/L and median (IQR) IL-6 levels were 3.3 (1.6–6.5) pg/mL. Pearson correlation between log-transformed CRP and log-transformed IL-6 was 0.21. There were 23.9% participants with prevalent hypertension, 18.9% with prevalent diabetes, and 10.7% with prevalent CVD. Characteristics of all study participants are shown by quintiles of dietary Mg in Table 1. Participants in the highest quintile of dietary Mg were more likely to be older and experienced a higher prevalence of hypertension, diabetes, and CVD compared to those in lower quintiles of Mg intake.

**Table 1:** Participant characteristics according to quintiles of total Mg intake.

Variable	Total	Dietary Mg Q1	Dietary Mg Q2	Dietary Mg Q3	Dietary Mg Q4	Dietary Mg Q5
N	1924	385	385	384	385	385
Median Total Mg (mg/day)*	283.1 (198.4–407.9)	204.1 (140.9–314.7)	268.4 (187.1–399.1)	299.0 (221.0–446.1)	310.3 (223.1–454.0)	322.4 (246.3–428.8)
N using supplements	441	7	26	53	104	251
Male (%)	39.2	45.7	40.5	41.7	44.7	23.4
Age (ys)**	40.3 (16.6)	31.1 (12.4)	35.3 (14.4)	39.1 (15.7)	45.0 (16.2)	50.9 (16.1)
Education (ys)**	12.3 (2.3)	11.8 (1.8)	12.0 (2.3)	12.1 (2.3)	12.6 (2.3)	13.0 (2.4)
BMI (kg/m^2^)**	31.5 (7.5)	31.1 (7.7)	31.8 (8.5)	32.0 (7.9)	31.5 (6.4)	31.2 (6.7)
Steps (per day)**	5907.0 (3992.7)	6431.6 (3901.4)	5894.6 (3770.2)	5781.9 (4114.1)	6110.0 (4344.3)	5322.3 (3736.3)
Smoking (%)						
Never	40.8	46.7	44.4	33.1	39.5	40.2
Ever	23.3	15.1	19.2	26.3	26.5	29.4
Current	35.9	38.2	36.4	40.6	34.0	30.4
Packs (per yr among ever/current smokers)**	11.3 (17.5)	7.1 (11.8)	9.5 (15.3)	10.3 (19.8)	14.2 (19.4)	15.1 (17.7)
Hypertension (%)	23.9	14.6	20.3	22.2	28.6	33.6
Diabetes (%)	18.9	11.2	13.0	19.1	25.8	25.7
CVD (%)	10.7	6.2	9.9	10.7	11.4	15.1
Calories (kcal/day)**	2431.8 (1334.3)	2564.2 (1321.6)	2721.2 (1404.2)	2773.3 (1502.3)	2345.5 (1183.4)	1755.7 (928.2)
Fiber (g/day)**	17.9 (11.4)	14.1 (8.5)	18.2 (10.8)	20.5 (12.5)	19.4 (11.9)	17.4 (11.7)
Folate (mcg/day)**	411.8 (244.7)	342.1 (211.6)	419.2 (230.7)	475.1 (286.2)	440.4 (248.7)	382.2 (218.1)
Total Fat (% of calories)**	38.3 (7.2)	38.2 (8.4)	38.8 (6.6)	39.2 (6.2)	38.7 (6.5)	36.8 (7.6)
Vegetable (servings/day)**	2.7 (2.1)	2.0 (1.5)	2.8 (2.0)	3.0 (2.3)	2.9 (2.2)	2.7 (2.3)
Fruit (servings/day)**	1.0 (0.9)	0.7 (0.6)	0.9 (0.7)	1.1 (0.8)	1.2 (0.9)	1.3 (1.1)
Median CRP (mg/L)*	3.7 (1.5–7.9)	3.1 (1.3–7.1)	3.5 (1.3–8.4)	4.3 (1.6–8.1)	3.8 (1.8–7.6)	3.9 (1.9–8.0)
Median IL-6 (pg/mL)*	3.3 (1.6–6.5)	3.2 (1.5–6.6)	3.3 (1.6–6.7)	3.7 (1.9–7.1)	3.1 (1.4–5.8)	3.3 (1.6–5.9)
Rs3740393, % genotype***						
G/G	82 (4.3)	21 (5.5)	11 (2.9)	19 (4.9)	14 (3.6)	17 (4.4)
C/G	540 (28.0)	120 (31.1)	117 (30.3)	102 (26.6)	84 (21.8)	117 (30.4)
C/C	964 (50.1)	207 (53.8)	212 (55.1)	190 (49.5)	194 (50.4)	161 (41.8)
Missing	338 (17.6)	37 (9.6)	45 (11.7)	73 (19.0)	93 (24.2)	90 (23.4)

*Median (IQR)

**Mean (SD)

***N (%)

Reported Mg intake was not associated with CRP or IL-6 (Table 2). We observed no interaction of reported Mg intake with rs3740393 on CRP (Table 3). However, we observed a significant interaction (p-interaction = 0.018) of reported Mg intake with rs3740393 on IL-6 (Table 4). Among participants with the C/C genotype, for every 1 SD higher in log-Mg, log-IL-6 was 0.04 (95% CI: -0.10 to 0.17) pg/mL higher. Among participants with the C/G genotype, the comparable change in log-IL-6 was 0.08 (95% CI: -0.21 to 0.05) pg/mL lower, and among those with the G/G genotype, log-IL-6 was 0.19 (95% CI: -0.38 to -0.01) pg/mL lower. The frequency of allele G at rs3740393 was 0.22. Participants missing genotype data were excluded from the Mg x genotype analyses.

**Table 2:** Association of log-Mg with log-biomarkers of inflammation *(estimates corresponding to 1 SD of log-Mg)*.

	log(CRP)		log(IL-6)	
	Estimate (95% CI)	P-value	Estimate (95% CI)	P-value
Model A*	0.01 (-0.10, 0.11)	0.906	-0.05 (-0.16, 0.06)	0.364
Model B**	0.05 (-0.07, 0.16)	0.433	-0.02 (-0.13, 0.09)	0.689

*Adjusted for age, sex, site, total calorie intake

**Adjusted for variables in Model A plus education, alcohol consumption, smoking, BMI, steps per day, hypertension, diabetes, CVD, and dietary intake of fiber, folate, % total fat, vegetable and fruits.

**Table 3:** Regression coefficients for the interaction of log-Mg and rs3740393 on log-CRP.

	log(CRP)	
	Estimate (95% CI)	P-value
Model A*	0.02 (-0.18, 0.23)	0.820
Model B**	0.03 (-0.14, 0.20)	0.703

*Adjusted for age, sex, site, total calorie intake

**Adjusted for variables in Model A plus education, alcohol consumption, smoking, BMI, steps per day, hypertension, diabetes, CVD, and dietary intake of fiber, folate, % total fat, vegetables and fruits, and SNPs associated with CRP (rs1205, rs3091244)

**Table 4:** Regression coefficients for the interaction of log-Mg and rs3740393 on log-IL-6 and the relationship between log-Mg and log-IL-6 for each rs3740393 genotype *(estimates corresponding to 1 SD of log-Mg)*.

	log(IL-6)				
	Estimate (95% CI)	P-value	Genotype	N (%)	Genotype Estimate (95% CI)
Model A*	0.22 (0.04, 0.40)	0.016	G/G	82 (5.2)	-0.24 (-0.42, -0.05)
			C/G	540 (34.0)	-0.11 (-0.24, 0.02)
			C/C	964 (60.8)	0.004 (-0.13, 0.14)
Model B**	0.22 (0.04, 0.40)	0.018	G/G	82 (5.2)	-0.19 (-0.38, -0.01)
			C/G	540 (34.0)	-0.08 (-0.21, 0.05)
			C/C	964 (60.8)	0.04 (-0.10, 0.17)

*Adjusted for age, sex, site, total calorie intake

**Adjusted for variables in Model A plus education, alcohol consumption, smoking, BMI, steps per day, hypertension, diabetes, CVD, and dietary intake of fiber, folate, % total fat, vegetable and fruits.

Analyses examining Mg intake with CRP or IL-6 that modeled Mg in quintiles produced similar results (S1 Table). Results were not meaningfully changed in analyses that excluded participants with prevalent diabetes or CVD (S2 Table). Sensitivity analyses that excluded participants with high levels of CRP or IL-6 (n = 259) produced similar results, but the interaction of Mg intake with rs3740393 on IL-6 was no longer significant (p-interaction = 0.599) (S3 Table).

There was no evidence of interaction of age or sex with reported intake of Mg on CRP or IL-6 (S4 Table). However, in exploratory analyses, associations of reported intake of Mg with CRP were modified by BMI (p-interaction = 0.0007) (S4 Table). Among participants with BMI of 25 kg/m^2^, for every 1 SD higher in log-Mg, log-CRP was 0.10 (95% CI: -0.01 to 0.22) mg/L higher (S5 Table). Comparable values were 0.01 (95% CI: -0.13 to 0.11) mg/L, and 0.12 (95% CI: -0.27 to 0.03) mg/L lower for a BMI of 35 and 45 respectively (S5 Table). This suggests that higher reported Mg intake was associated with lower CRP among participants with higher BMI.

Results of analyses that assessed the associations of the selected SNPs with CRP and IL-6 are shown in S6 Table. We observed no association of SNP rs3740393 with either CRP or IL-6. SNP rs1205 was significantly associated with CRP (p-value = 0.004), suggesting that log-CRP levels were 0.12 mg/L higher with each additional copy of the C allele. SNP rs3091244 was also significantly associated with CRP (p = 0.002), suggesting that log-CRP levels were 0.15 mg/L higher with each additional copy of the A allele. Neither SNP rs1205 nor rs3091244 were associated with IL-6.

## Discussion

In this cross-sectional analysis of AI participants from the SHFS, rs3740393 modified the association of reported Mg intake with IL-6 (but not CRP), after adjustment for factors known to be associated with Mg intake and inflammation, including dietary factors, prevalent diabetes, and prevalent CVD. These findings suggest that genetic factors related to Mg transport and metabolism may influence the association of Mg with inflammation in a population at high risk for inflammatory-related chronic diseases (i.e., CVD and diabetes). However, the detected association appears to be driven by a subset of participants with higher CRP or IL-6 levels, perhaps indicating the influence of acute inflammation, rather than genetics.

The findings reported herein do not readily support the notion that higher dietary Mg intake is associated with lower levels of CRP, as is suggested by previous observational studies [3, 4, 12, 28, 29]. In particular, our findings are not consistent with results from the Women’s Health Initiative (WHI) [3] or the National Health and Nutrition Examination Survey (NHANES) 1999–2000 [28], both of which reported that Mg intake is inversely associated with CRP concentrations in samples of healthy adults. However, the SHFS sample was smaller compared to both the WHI [3] and NHANES [28] samples and may have lacked power to detect this association. In addition, CRP levels among SHFS participants were higher when compared to participants in WHI or NHANES. Mean CRP was 5.1 mg/L in WHI [3] compared to 6.6 mg/L in SHFS, and median CRP was 2.0 mg/L in NHANES [28] compared to 3.7 in SHFS. Mean age was also lower in SHFS compared to many of the other observational studies [3, 4, 12], while average BMI [4, 12, 29] and percent of current smokers [3, 4, 12, 28] were higher in the SHFS compared to other studies. Therefore, it is possible that differences in power, underlying CRP, baseline age, BMI, and smoking status in part explain these conflicting findings.

While individual randomized controlled trials show no effect of oral Mg supplementation on CRP [6–10], a meta-analysis including some of these trials indicated that supplementation reduces CRP [11]. In general, trials varied with regards to amount and duration of supplementation. However, trials incorporated in the meta-analysis included participants with Mg deficiency [30], hypomagnesemia [7, 31], those undergoing coronary artery bypass surgery [32], and hemodialysis patients [9], characteristics that are not representative of the SHFS population.

Previous studies that have examined the association of reported Mg intake with IL-6 are inconsistent [3, 12, 13, 29]. To our knowledge, no studies have assessed the potential effect of genes associated with Mg transport and metabolism on the association of reported Mg intake with IL-6. Our findings build upon previous work and suggest that higher intake of Mg may be associated with IL-6 among individuals with one or more copies of the G allele at rs3740393. This SNP is located in *CNNM2*, a gene which encodes a membrane protein necessary for Mg transport. Previous studies have found that the allele corresponding to C in the SHFS population is associated with lower serum Mg [16, 33]. Thus, this allele may reduce the power to demonstrate efficacy of dietary Mg, since dietary Mg would not be well absorbed and thus less available as serum Mg. It is possible that we observed a significant interaction of Mg intake and rs3740393 on IL-6, but not on CRP because the role of IL-6 is further upstream in the inflammatory pathway compared to CRP [3]. However, as sensitivity analyses excluding participants in an acute inflammatory state indicated no significant interaction of dietary Mg and rs3740393 on CRP or IL-6, the significant interaction on IL-6 seen in main analyses may be driven by higher levels of inflammation or be a result of more power. Additional research is needed to better understand the interplay of SNPs, Mg, and inflammation.

This study has many strengths including the availability of genetic data to allow examination of the interplay of relevant SNPs, dietary Mg, and inflammation. The SHFS is a large, family-based sample, and used validated instruments to collect data on a wide array of factors, allowing for well-measured assessments of important covariates. Additionally, this study focused on AIs, a minority population with a high burden of diabetes and CVD.

This study also has limitations. As the SHFS only has a single measure of Mg intake, analyses were cross-sectional, and we are unable to infer temporality. Similar to other epidemiological studies that have investigated the relationship of dietary Mg intake with markers of inflammation [3, 4, 12, 28, 29], total dietary Mg was calculated based on food and supplement use [3, 4, 12, 13, 28, 29]. We were unable to account for drinking water, another potential source of dietary Mg [34], in our analyses; SHFS participants obtain drinking water from a wide variety of sources (for example, municipal water supplies, commerically available bottled water, private wells), and it was not feasible to sample the drinking water of the 1,924 participants that comprised this large epidemiolgical cohort. However, on average, drinking water accounts for approximately 2.4% of the RDA for magnesium intake for adults in the USA [34, 35], representing a significantly smaller portion of total dietary Mg than Mg derived from food and dietary supplements. Additionally, this study focused on self-reported intake of dietary Mg, and we did not consider Mg in serum or red blood cells as part of the analysis plan because this data is unavailable in the SHFS. However, about 99% of Mg in humans is in bone and intracellular soft tissue, and Mg in serum or red blood cells are poor predictors of intracellular magnesium concentration [36]. As previous studies have consistently reported poor correlation between serum Mg and dietary Mg (r = 0.04), serum Mg is not a robust biomarker of dietary intake [37]. Finally, genetic information for AIs from the SHFS is currently restricted to those SNPs included on the MetaboChip, which were not optimally selected to include variants specific to North American Indigenous ancestry.

In conclusion, our results indicate that dietary intake of Mg is not associated with CRP (irrespective of genotype), but that Mg intake may be associated with lower IL-6 among those with two copies of the G allele at rs3740393. Future research is necessary to replicate this finding, and to examine other Mg-related genes that may influence associations of Mg intake with inflammation.

## Supporting information

S1 Fig Participant flowchart. (PDF)

S1 Table Regression coefficients for the associations of quintiles of dietary Mg and log-biomarkers of inflammation. (DOCX)

S2 Table Association of log-Mg with log-biomarkers of inflammation among participants without diabetes or CVD. (DOCX)

S3 Table Regression coefficients for the interaction of log-Mg and rs3740393 on log-biomarkers of inflammation among participants with CRP < 16 mg/L and IL-6 < 16 pg/mL. (DOCX)

S4 Table Regression coefficients for the interactions of log-Mg and age, sex and BMI on log-biomarkers of inflammation. (DOCX)

S5 Table Relationship of log-Mg with log-CRP at different BMI. (DOCX)

S6 Table Regression coefficients for the associations of SNPs with log-CRP and log-IL-6. (DOCX)
