# Supplementary figures and images for: Dietary magnesium, C-reactive protein and interleukin-6: The Strong Heart Family Study
Source: PLoS One. 2023 Dec 21;18(12):e0296238. doi: 10.1371/journal.pone.0296238 (PMC10734955; doi:10.1371/journal.pone.0296238)

**Supplementary Figure 1: Participant Flowchart**

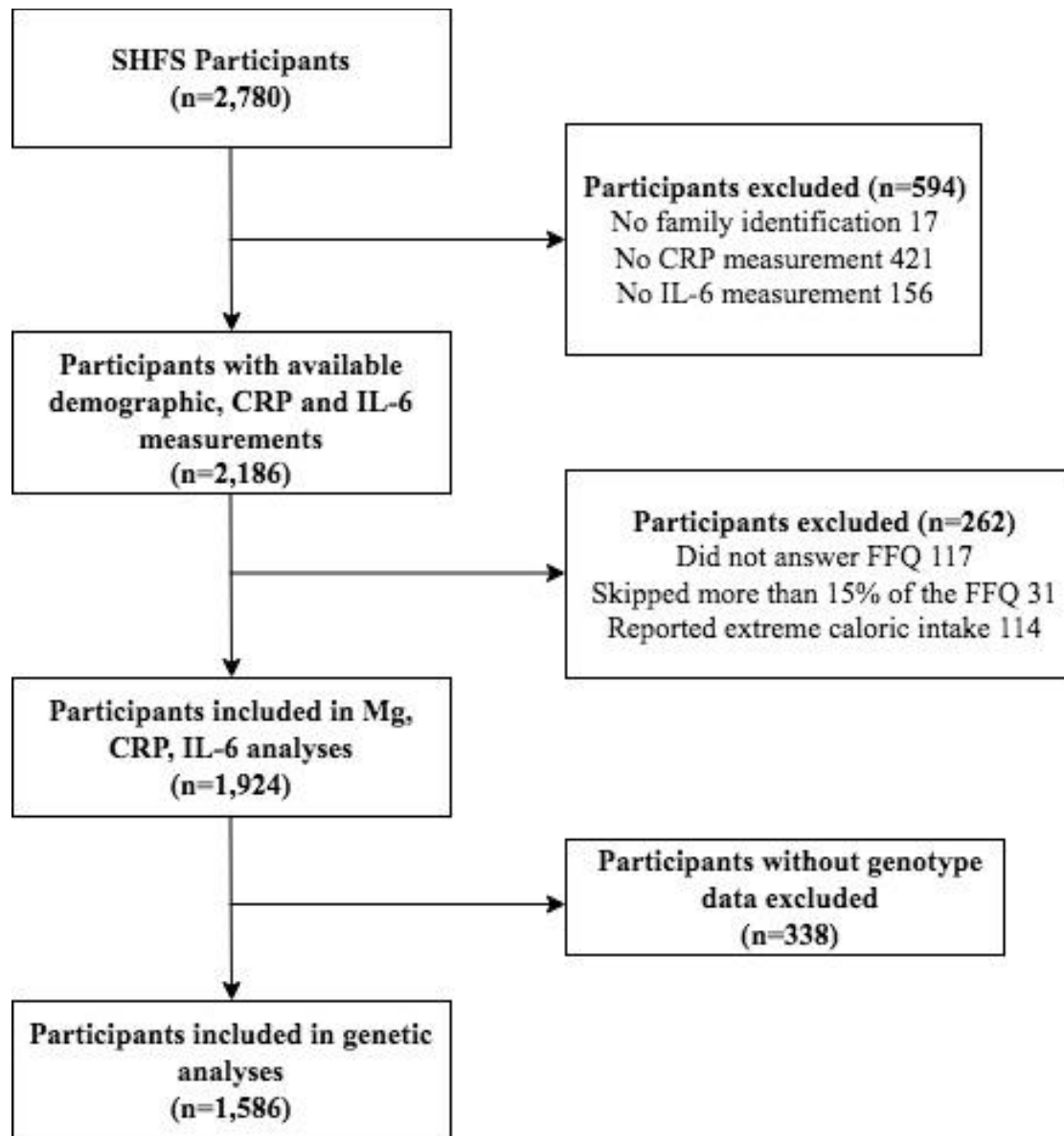

Supplement: S1 Fig — (PDF) [file pone.0296238.s001.pdf]
