# Supplementary material for: Dietary magnesium, C-reactive protein and interleukin-6: The Strong Heart Family Study
Source: PLoS One. 2023 Dec 21;18(12):e0296238. doi: 10.1371/journal.pone.0296238 (PMC10734955; doi:10.1371/journal.pone.0296238)
Supplement: S1 Table — (DOCX) [file pone.0296238.s002.docx]

**Supplementary Table 1: Regression coefficients for the associations of quintiles of dietary Mg and log-biomarkers of inflammation** *(Q1 used as reference)*

|  |  | **log(CRP)** | | **log(IL-6)** | |
| --- | --- | --- | --- | --- | --- |
|  | Total Mg Quartile | Estimate (95% CI) | P for trend | Estimate (95% CI) | P for trend |
| Model A* | Q2 | 0.03 (-0.13, 0.20) | 0.031 | 0.002 (-0.19, 0.20) | 0.259 |
|  | Q3 | 0.02 (-0.14, 0.18) |  | 0.11 (-0.12, 0.33) |  |
|  | Q4 | -0.06 (-0.22, 0.10) |  | -0.18 (-0.39, 0.04) |  |
|  | Q5 | -0.19 (-0.36, -0.01) |  | -0.08 (-0.30, 0.16) |  |
| Model B** | Q2 | -0.02 (-0.18, 0.14) | 0.075 | -0.02 (-0.21, 0.18) | 0.443 |
|  | Q3 | -0.04 (-0.19, 0.10) |  | 0.09 (-0.13, 0.31) |  |
|  | Q4 | -0.10 (-0.27, 0.08) |  | -0.17 (-0.40, 0.06) |  |
|  | Q5 | -0.18 (-0.38, 0.02) |  | -0.04 (-0.28, 0.20) |  |

*Adjusted for age, sex, site, total calorie intake

**Adjusted for variables in Model A plus education, alcohol consumption, smoking, BMI, steps per day, hypertension, diabetes, CVD, and dietary intake of fiber, folate, % total fat, vegetable and fruits.
