# Supplementary material for: Dietary magnesium, C-reactive protein and interleukin-6: The Strong Heart Family Study
Source: PLoS One. 2023 Dec 21;18(12):e0296238. doi: 10.1371/journal.pone.0296238 (PMC10734955; doi:10.1371/journal.pone.0296238)
Supplement: S2 Table — (DOCX) [file pone.0296238.s003.docx]

**Supplementary Table 2: Association of log-Mg with log-biomarkers of inflammation among participants without diabetes or CVD (n=1,450)** *(estimates corresponding to 1 SD of log-Mg)*

|  | **log(CRP)** | | **log(IL-6)** | |
| --- | --- | --- | --- | --- |
|  | Estimate (95% CI) | P-value | Estimate (95% CI) | P-value |
| Model A* | 0.04 (-0.09, 0.17) | 0.536 | -0.04 (-0.17, 0.09) | 0.506 |
| Model B** | 0.04 (-0.09, 0.18) | 0.537 | -0.001 (-0.14, 0.14) | 0.986 |

*Adjusted for age, sex, site, total calorie intake

**Adjusted for variables in Model A plus education, alcohol consumption, smoking, BMI, physical exercise, hypertension, diabetes, CVD, fiber, folate, % total fat, vegetable and fruit intake
