# Supplementary material for: Dietary magnesium, C-reactive protein and interleukin-6: The Strong Heart Family Study
Source: PLoS One. 2023 Dec 21;18(12):e0296238. doi: 10.1371/journal.pone.0296238 (PMC10734955; doi:10.1371/journal.pone.0296238)
Supplement: S3 Table — (DOCX) [file pone.0296238.s004.docx]

**Supplementary Table 3: Regression coefficients for the interaction of log-Mg and rs3740393 on log-biomarkers of inflammation among participants with CRP < 16 mg/L and IL-6 < 16 pg/mL (n=1,327)**

|  | **log(CRP)** | | **log(IL-6)** | |
| --- | --- | --- | --- | --- |
|  | Estimate (95% CI) | P-value | Estimate (95% CI) | P-value |
| Model A* | 0.01 (-0.19, 0.20) | 0.955 | 0.05 (-0.11, 0.21) | 0.565 |
| Model B** | 0.03 (-0.13, 0.18) | 0.738 | 0.05 (-0.12, 0.21) | 0.599 |

*Adjusted for age, sex, site, total calorie intake

**Adjusted for variables in Model A plus education, alcohol consumption, smoking, BMI, steps per day, hypertension, diabetes, CVD, and dietary intake of fiber, folate, % total fat, vegetables and fruits. For the outcome log(CRP), additional adjustments made for SNPs associated with CRP (rs1205, rs3091244)
