# Supplementary material for: Dietary magnesium, C-reactive protein and interleukin-6: The Strong Heart Family Study
Source: PLoS One. 2023 Dec 21;18(12):e0296238. doi: 10.1371/journal.pone.0296238 (PMC10734955; doi:10.1371/journal.pone.0296238)
Supplement: S4 Table — (DOCX) [file pone.0296238.s005.docx]

**Supplementary Table 4: Regression coefficients for the interactions of log-Mg and age, sex and BMI on log-biomarkers of inflammation**

|  | **log(CRP)** | | **log(IL-6)** | |
| --- | --- | --- | --- | --- |
|  | Estimate (95% CI) | P-value | Estimate (95% CI) | P-value |
| Age | -0.005 (-0.01, 0.001) | 0.091 | -0.005 (-0.01, 0.002) | 0.136 |
| Sex | -0.002 (-0.20, 0.19) | 0.981 | -0.03 (-0.27, 0.21) | 0.834 |
| BMI | -0.02 (-0.03, -0.01) | 0.0007 | -0.01 (-0.02, 0.003) | 0.124 |

Adjusted for age, sex, site, total calorie intake, education, alcohol consumption, smoking, BMI, steps per day, hypertension, diabetes, CVD, and dietary intake of fiber, folate, % total fat, vegetable and fruits.
