# Supplementary material for: Dietary magnesium, C-reactive protein and interleukin-6: The Strong Heart Family Study
Source: PLoS One. 2023 Dec 21;18(12):e0296238. doi: 10.1371/journal.pone.0296238 (PMC10734955; doi:10.1371/journal.pone.0296238)
Supplement: S5 Table — (DOCX) [file pone.0296238.s006.docx]

**Supplementary Table 5: Relationship of log-Mg with log-CRP at different BMI** *(estimates corresponding to 1 SD of log-Mg)*

| **log(CRP)** | | |
| --- | --- | --- |
| BMI | Estimate (95% CI) | P-value for interaction |
| 25 | 0.10 (-0.01, 0.22) | 0.0007 |
| 30 | 0.05 (-0.06, 0.16) |  |
| 35 | -0.01 (-0.13, 0.11) |  |
| 40 | -0.06 (-0.20, 0.07) |  |
| 45 | -0.12 (-0.27, 0.03) |  |

Adjusted for age, sex, site, total calorie intake, education, alcohol consumption, smoking, steps per day, hypertension, diabetes, CVD, and dietary intake of fiber, folate, % total fat, vegetable and fruits.
