# Supplementary material for: Dietary magnesium, C-reactive protein and interleukin-6: The Strong Heart Family Study
Source: PLoS One. 2023 Dec 21;18(12):e0296238. doi: 10.1371/journal.pone.0296238 (PMC10734955; doi:10.1371/journal.pone.0296238)
Supplement: S6 Table — (DOCX) [file pone.0296238.s007.docx]

**Supplementary Table 6: Regression coefficients for the associations of SNPs with log-CRP and log-IL-6** *(estimates corresponding to 1 copy of the risk allele)*

|  | **log(CRP)** | | **log(IL-6)** | |
| --- | --- | --- | --- | --- |
|  | Estimate (95% CI) | P-value | Estimate (95% CI) | P-value |
| rs3740393 | 0.03 (-0.08, 0.13) | 0.609 | 0.07 (-0.07, 0.20) | 0.333 |
| rs1205 | 0.12 (0.04, 0.20) | 0.004 | 0.02 (-0.07, 0.11) | 0.688 |
| rs3091244 | 0.15 (0.06, 0.25) | 0.002 | -0.01 (-0.10, 0.09) | 0.889 |

Adjusted for age and sex
